# Spectroscopic Evidence for a Covalent Sigma Au–C Bond on Au Surfaces Using ^13^C Isotope Labeling

**DOI:** 10.1021/jacsau.0c00108

**Published:** 2021-02-23

**Authors:** Huaiguang Li, Gabriel Kopiec, Frank Müller, Frauke Nyßen, Kyoko Shimizu, Marcel Ceccato, Kim Daasbjerg, Nicolas Plumeré

**Affiliations:** † Center for Electrochemical Sciences (CES), Faculty of Chemistry and Biochemistry, Ruhr University Bochum, Universitätsstr. 150, D-44780 Bochum, Germany; ‡ Interdisciplinary Nanoscience Center/Department of Chemistry, Aarhus University, Gustav Wieds Vej 14, 8000 Aarhus C, Denmark; § Campus Straubing for Biotechnology and Sustainability, Technical University Munich, Schulgasse 22, 94315 Straubing, Germany

**Keywords:** Au−C bond, isotope labeling, SERS, solid-state NMR, Au nanoparticles, aryldiazonium salts

## Abstract

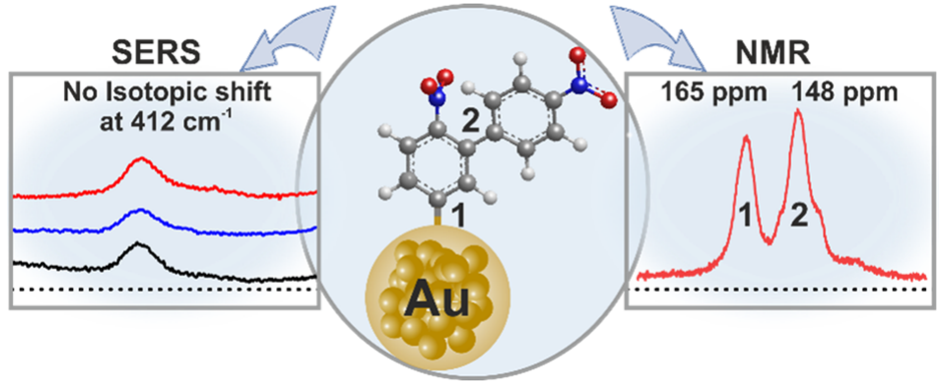

The Au–C linkage has been demonstrated as a robust interface for coupling thin organic films on Au surfaces. However, the nature of the Au–C interaction remains elusive up to now. Surface-enhanced Raman spectroscopy was previously used to assign a band at 412 cm^–1^ as a covalent sigma Au–C bond for films generated by spontaneous reduction of the 4-nitrobenzenediazonium salt on Au nanoparticles. However, this assignment is disputed based on our isotopic shift study. We now provide direct evidence for covalent Au–C bonds on the surface of Au nanoparticles using ^13^C cross-polarization/magic angle spinning solid-state NMR spectroscopy combined with isotope substitution. A ^13^C NMR shift at 165 ppm was identified as an aromatic carbon linked to the gold surface, while the shift at 148 ppm was attributed to C–C junctions in the arylated organic film. This demonstration of the covalent sigma Au–C bond fills the gap in metal–C bonds for organic films on surfaces, and it has great practical and theoretical significance in understanding and designing a molecular junction based on the Au–C bond.

## Introduction

Direct anchoring of organic molecules to a metal or metallic nanoparticles (NPs) and nanoclusters is fundamental in various fields,^1−4^ such as molecular electronics, biomedical tools engineering, and electrochemical devices. In addition, single-molecule grafting techniques enable the manipulation and processing of single units of charge transport, which is an essential step toward creating quantum-effect devices.^5^ By now, various anchoring groups (thiol,^6,7^ pyridine,^8^ amine,^9,10^ isocyanide,^11^ nitrile,^12^ and carbene^13^) have been exploited to attach molecules to the surfaces of substrates. Of particular interest has been the exploration of the Au–C linkage.^14^ In addition to bonding carbene^4,13^ or terminal alkynyl groups^15,16^ directly to Au, the Au–C linkage has been realized by cleaving trimethyltin terminal groups^14^ and silyl groups.^17^ Perhaps the most prominent pathway to introduce organic molecules with this junction involves aryldiazonium ions^18,19^ because of the ease by which various functional groups on the aromatic ring can be introduced. The considerable interest in Au–C bonding is due in part to its significant robustness and excellent ambient, thermal, and chemical stability.^13,18,20^ In fact, by mechanically breaking the junction, it was shown that the Au–C bonding results in a more robust junction than the corresponding Au–N bonding obtained if the same molecule was connected using amine linkers. In addition, Au–C bonded single-molecule junctions exhibit a much larger conductance than most other linkages.^14,21^

In spite of the intense research interest in the Au–C bonding on Au surfaces, direct experimental evidence of its existence is still missing to support the calculations of its nature.^22,23^ X-ray photoelectron spectroscopy (XPS) is typically applied to investigate interactions for modified metal surfaces, including Au. Laforgue et al.^24^ reported the details of the Au 4f core level spectrum from diazonium grafting and indicated that the linkage of Au–O–C is unlikely, because the binding energies for the Au 4f peaks correspond exactly to metallic and not oxidized gold. However, definite evidence for a covalent linkage (Au–C and/or Au–N=N–C) could not be obtained, although the presence of the functional groups associated with the corresponding diazonium species at the surface was confirmed. Similar conclusions were reached in subsequent studies,^25^ in which Bélanger^26^ and Gooding^27^ were unable to observe clear binding energy components corresponding to Au–C. Presumably, the missing evidence from XPS can be attributed to the low polarity of the Au–C bond, which makes its signal merge with those of the C–C bonds.^28^

Until now, the strongest evidence of a Au–C bond from Au NPs modified with nitrobenzenediazonium salts was presented by using surface-enhanced Raman scattering (SERS), where a weak SERS band at 412 cm^–1^ was assigned to the Au–C stretching vibration.^29^ Similar results were observed from binding acetylene and alkyl chains to Au surfaces,^16,30,31^ although the assignments of the corresponding SERS bands (at 432, 397, and 387 cm^–1^) still need to be validated. Time-of-flight secondary ion mass spectrometry (ToF-SIMS) suggested the presence of fragments containing Au–C,^25^ but these were only a few of the possible structures. In particular, the large number of signals typically observed in ToF-SIMS spectra of organic films prepared by aryldiazonium salts reduction weakens the assignment validity. Finally, extended X-ray absorption fine structure analysis only yielded indirect evidence of the Au–C bond from terminal alkynes on Au clusters.^32^

**Scheme 1:**
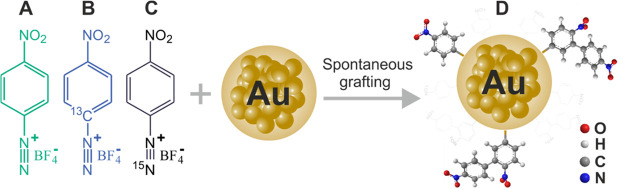
Isotopically Labeled Aryldiazonium Salts Used for Raman, NMR, and Mass Spectrometry Investigations (A) 4-Nitrobenzenediazonium tetrafluoroborate (NBD), (B) 4-nitro-[1-^13^C]-benzenediazonium tetrafluoroborate (^13^C NBD), and (C) ^15^N labeling 4-nitrobenzenediazonium tetrafluoroborate (^15^N NBD). (D) Proposed structures of aryl moieties attached on the Au NPs after spontaneous grafting of the aryldiazonium salts.

In this work, we utilized ^13^C cross-polarization/magic angle spinning solid-state NMR (^13^C CP/MAS ssNMR) combined with ToF-SIMS to unequivocally demonstrate the presence of Au–C bonds on Au surfaces modified by spontaneous reduction of the 4-nitrobenzenediazonium salt (NBD, Scheme 1). This diazonium salt was chosen, since it is widely studied for various surface modifications, and its grafting process is a relatively easy operation.^18^ In addition to NBD, we synthesized its ^13^C and ^15^N labeled derivatives (^13^C NBD and ^15^N NBD, Scheme 1, Supporting Information) and subsequently grafted those onto Au NPs by spontaneous reaction under identical conditions. The reason for these specific isotope labelings is that it is the ^13^C nuclei in the ipso position to the diazonium group that are expected to be involved in the bonding to the Au surface, and potentially, the ^15^N nuclei in the diazonium functionality could do the same.

An advantage of the NMR technique is that it probes the local atomic environment, allowing for structural identification without the need for long-range order.^33^ For example, the assignment of the Au–C bond in organometallic Au complexes is routinely performed via ^13^C NMR spectroscopy under homogeneous conditions, exploiting that the signal for the C nucleus in the ipso position to the Au is strongly downfield shifted.^34,35^ Here, we provide strong evidence of the Au–C bond on the basis of a signal appearing at 165 ppm. In addition to revealing the presence of the Au–C bond between the aryl layer and the Au substrate, the proposed methodology also provides a straightforward characterization of the multilayer structure of the film. ToF-SIMS measurements are used to validate these findings using both ordinary and isotopically labeled diazonium salts.

## Results and Discussion

### SERS

**Figure 1:**
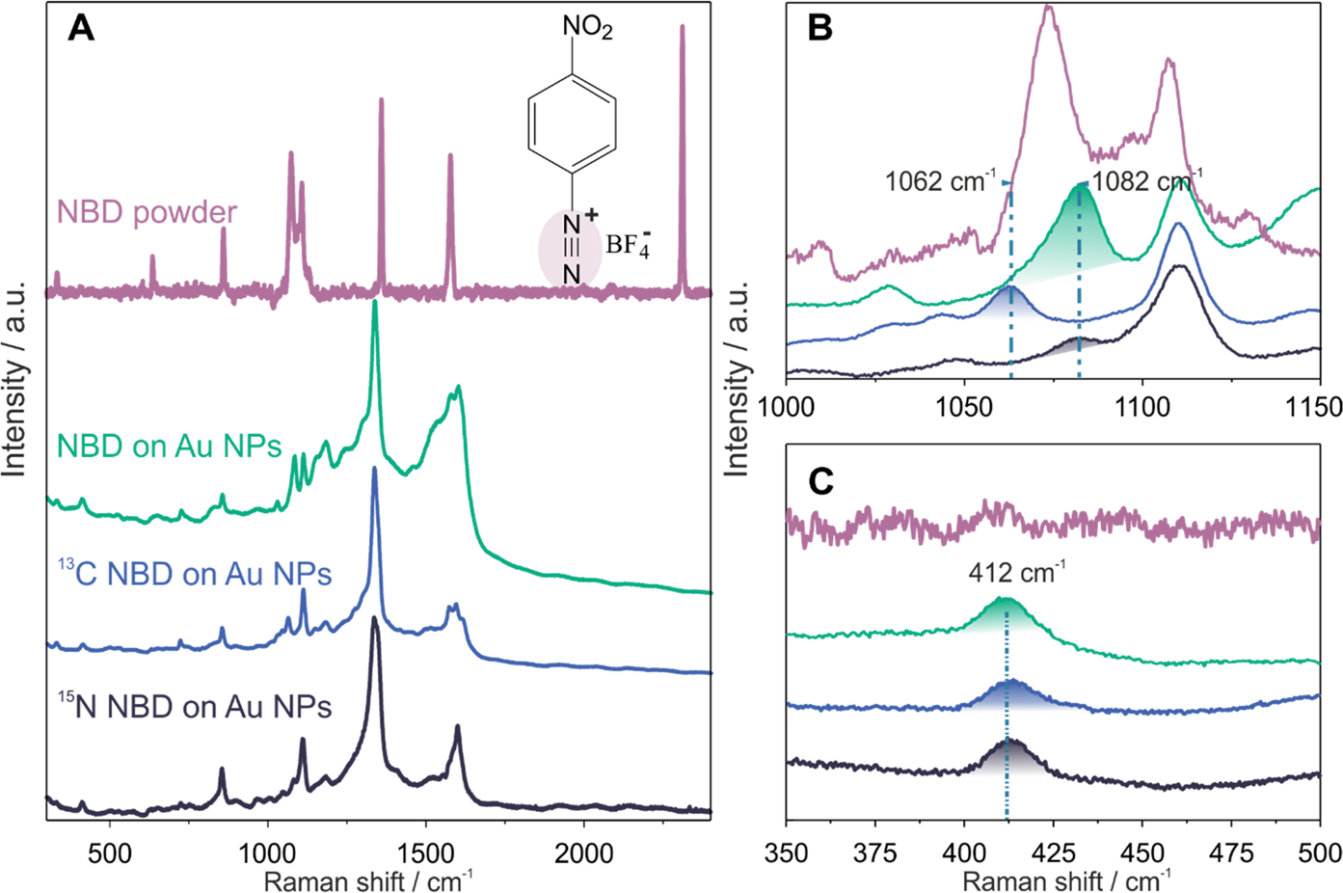
Raman spectra of pure 4-nitrobenzenediazonium tetrafluoroborate (NBD) powder (brown), NBD-modified Au NPs (green), ^13^C NBD-modified Au NPs (blue), and ^15^N NBD-modified Au NPs (black). (A) Full Raman spectra, 300–2400 cm^–1^; (B) zoom-in spectra range from 1000 to 1150 cm^–1^, and (C) zoom-in spectra range from 350 to 500 cm^–1^ derived from (A). The spectra were recorded experimentally at a wavelength of 661 nm as detailed in the Methods section.

First, the isotopic Raman shift was revealed by recording spectra of NBD and ^15^N NBD with bands assigned to N≡N stretching vibrations showing up at 2310 and 2277 cm^–1^, respectively (Figures 1A and S1). The difference of 33 cm^–1^ is caused by the difference in mass between the ^14^N and ^15^N isotopes. Noticeably, these two bands are absent after grafting NBD/^15^N NBD on the Au NPs, which is consistent with the loss of the dinitrogen triple-bond groups.^2,18^ Thus, unspecific adsorption of the unreacted diazonium salts on Au NPs does not take place. Also, high-resolution ^1^H NMR analysis shows that no reaction is proceeding for NBD dissolved in a CD_3_CN/deionized water mixture for 24 h at room temperature in the absence of Au NPs (Figure S2). This suggests that NBD grafting does not involve a homogeneous reaction followed by the subsequent adsorption on the Au NPs.

The differences of wavenumber caused by ^13^C enrichment after grafting onto Au NPs are shown in the bands at 1082 and 1062 cm^–1^ (Figure 1B). These are characteristic of CH in-plane bending coupled with a C–N stretch. The corresponding bands at 1599 and 1584 cm^–1^ are assigned to a ring stretch (Figure S3). The presence of these aryl-specific bands and the corresponding isotopic shifts demonstrate that isotopic substitution is potentially useful to examine the Au–C bond originating from the grafting of aryldiazonium salts onto Au NPs.

Density-functional theory (DFT) calculation predicted that covalent Au–C σ bonds on the Au surface should display a stretching vibration band at 412 cm^–1^.^25,29^ This signal was observed as a weak band at 412 cm^–1^ in the spectrum of NBD-modified Au NPs in a previous study,^29^ and it is confirmed in the present study (Figure 1C). However, we notice that this band is also found in the spectrum of the Au NPs modified with ^13^C NBD without any observed shift. The isotope exchange should theoretically shift the band in the SERS spectra from 412 to 395 cm^–1^ for the ^13^C enriched sample according to the diatomic model. Similar results regarding isotope shift were demonstrated from grafting azobenzene on ^13^C enriched graphite. The calculation based on the diatomic model for the C–C stretch band on a graphite surface was very close to the experimental observations.^36^ This strongly suggests that the carbon atom initially in the ipso position to the diazonium group is not involved in the vibration observed at 412 cm^–1^ for the modified Au NPs. This does not invalidate the DFT computation for Au–C vibrations, but it does demonstrate that another unassigned vibration coincides with this wavenumber. The Au–C vibration is expected to result in low-intensity bands due to the low polarity of this bond and may, hence, remain undetected by SERS. Notably, the spectrum of ^15^N NBD-modified Au NPs is almost identical to the one of the NBD-modified Au NPs, demonstrating that either the dinitrogen groups are fully lost as dinitrogen during grafting, or the bands involving ^15^N atoms are too weak to be detected. Therefore, the presence of the band at 412 cm^–1^ without an isotopic shift in the spectrum of Au NPs modified with ^15^N NBD also excludes the possibility of this band being related to Au–N=N–aryl vibrations.^37^

### ^13^C CP/MAS ssNMR

**Figure 2:**
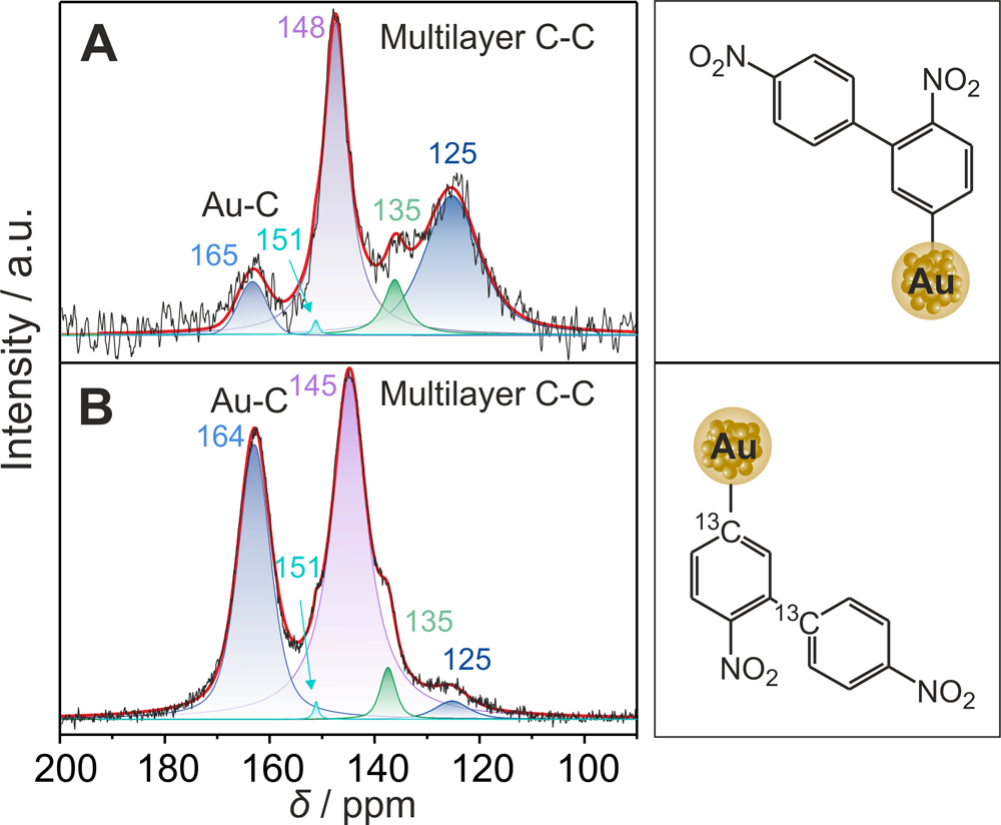
^13^C CP/MAS ssNMR spectra of (A) NBD-modified Au NPs and (B) ^13^C NBD-modified Au NPs with the proposed structures shown in the column to the right. NMR spectra were fitted using the Dmfit software to give five deconvoluted Gaussian–Lorentzian signals (shaded in colors).^31^ The black and red lines are the measured and fitted spectra, respectively.

Figure 2A shows the spectrum of NBD-modified Au NPs, revealing a broad resonance between 135 and 120 ppm along with a relatively sharp peak at 148 ppm and a broad band at 165 ppm. Deconvolution of the spectrum reveals five bands in total, with those at 125, 135, and 151 ppm within the expected range of chemical shifts for the meta, ortho, and para positions of aromatic carbon atoms. These are fully consistent with the solid-state and liquid ^13^C NMR spectra of NBD (Figure S4). In contrast, the assignments of 148 and 165 ppm, as two new bands, need further clarification. In particular, the signal at 165 ppm is of low field compared to the chemical shifts of aromatic carbons in organic molecules containing the nitrophenyl moiety (see below for exceptions to this rule).

Next, the ^13^C CP/MAS ssNMR spectrum of ^13^C NBD-modified Au NPs was recorded to decipher the new bands (Figure 2B). The most profound feature is the extremely high intensities of the signals at 148 and 165 ppm, which render the other aromatic signals (125, 135, and 151 ppm) almost invisible. Yet, these peak components as observed for the NBD-modified Au NPs could still be deconvoluted. There is a slight upfield shift, respectively, in the 165 ppm peak by 1 ppm and in the 148 ppm peak by 3 ppm, which can be attributed to experimental deviations. This observation infers that 148 and 165 ppm are both related to the presence of the isotope substituted ^13^C atoms at the ipso position, which is fully consistent with the NBD grafting mechanism where these carbon atoms are the ones suggested to form the bond with the substrate.^18^ Furthermore, an analogy can be drawn to organometallic complexes, in that the high-resolution ^13^C NMR spectra of an *N*-heterocyclic carbene (NHC)-functionalized Au cluster,^4^ (NHC)Au(I)–R,^35^ and various aryl–AuPPh_3_ complexes^34^ demonstrate that sigma Au–C covalent bonds yield low-field chemical shifts (209–170 ppm). Similarly, the Au–C bond resulting from aryldiazonium salt grafting onto Au NPs is expected to shift downfield because of the strong deshielding effect of Au on the C nuclei.

To exclude other bond types but Au–C as the origin of the 165 ppm peak, we need to consider potential side products from the reactions of NBD during grafting.^38^ In theory, 4-nitrophenol is such a candidate, since the carbon in ipso position to the −OH group would display a chemical shift (164.6 ppm; Table S1) in accordance with the one observed. Since the SERS band for the O–H bending vibration overlaps with the band of the C–H in plane (i.p.) bend for the samples of 4-nitrophenol and 4-nitrophenol mixed with Au NPs, the C–O stretch was chosen to identify phenolic moieties. The Raman spectra of both NBD and ^13^C NBD-modified Au NPs (Figure 2B) do not display any band that can be reasonably attributed to a C–O stretch (Figure S5). This result was further confirmed by Fourier transform infrared spectroscopy (FTIR), in which the signal assigned to the stretching vibration of the phenolic hydroxyl was absent between 3400 and 2800 cm^–1^ (Figure S6). Therefore, the presence of nitrophenolic compounds, including polymeric systems, on the Au NPs modified by reactions with NBD can be ruled out. Other potential organic side products from NBD such as (*E*)-4,4′-dinitroazobenzene would display a ^13^C NMR shift of the carbons ipso to the azo group in the range of 155.5–152 ppm.^39^ This range is far too high field to account for the signal at 165 ppm.

The second signal observed at 148 ppm indicates the formation of a second bonding interaction via the ipso carbon atoms in the NBD-grafted film. The nature of this multilayered film arising from further arylation of the nitrobenzene rings via an aryl radical mechanism has been identified elsewhere.^29,40,41^ It is generally difficult to control multilayer formation from the reduction of diazonium salts,^18,42−44^ and its reproducibility may fluctuate between different samples, even by using the same ratio of Au NPs and diazonium molecules. This is one of the possible reasons for the slight shift of this band between different batches of the modified Au NPs. Moreover, the band at 148 ppm is fully consistent with the one reported in the literature for 4-nitro-2′-nitrobiphenyl,^45^ which is a molecular analogue for the postulated structure of the organic film on the Au surface. Therefore, the band at 148 ppm is assigned as the C–C bond in the multilayer junction.

### ToF-SIMS

**Figure 3:**
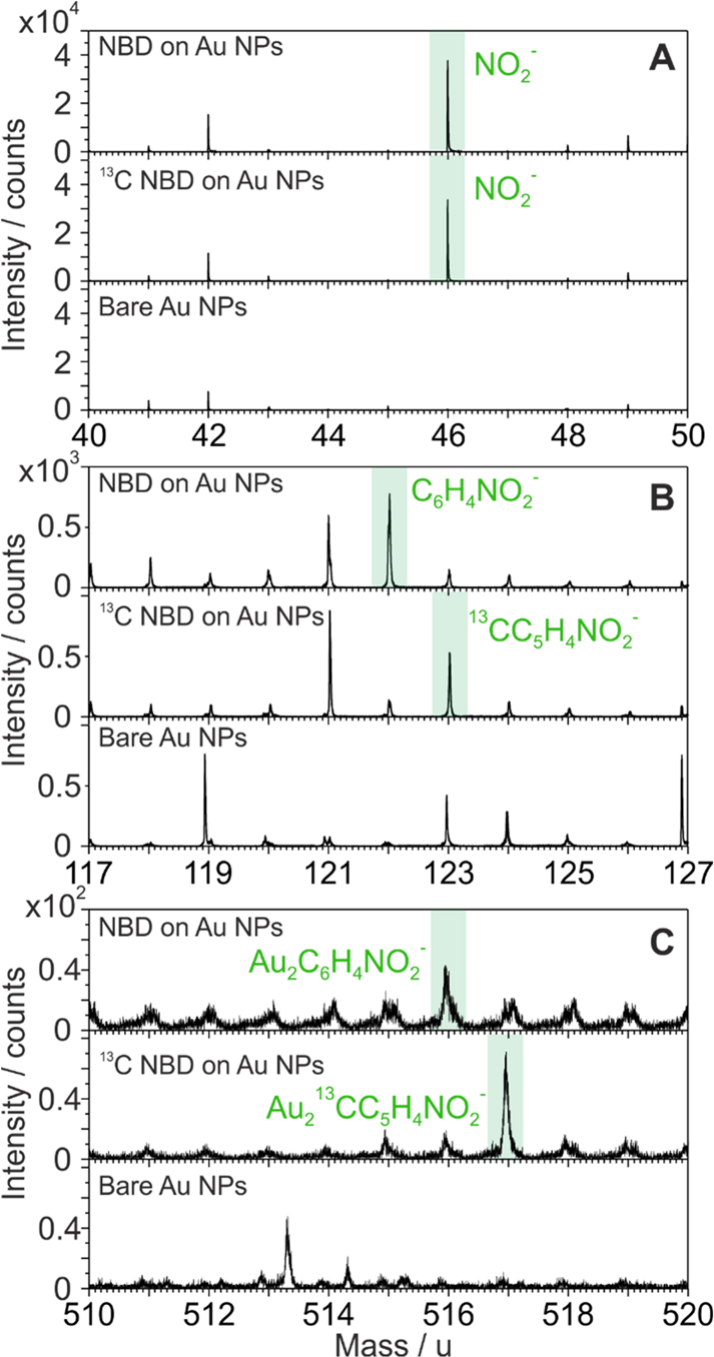
Negative high-resolution ToF-SIMS spectra of bare Au (background), NBD-modified Au NPs, and ^13^C NBD-modified Au NP samples pertaining to fragments of (A) NO_2_^–^, (B) C_6_H_4_NO_2_^–^ and ^13^CC_5_H_4_NO_2_^–^, and (C) Au_2_C_6_H_4_NO_2_^–^ and Au_2_^13^CC_5_H_4_NO_2_^–^.

To substantiate this interpretation, ToF-SIMS experiments were subsequently performed on the NBD and ^13^C NBD-grafted Au NPs. The mass difference resulting from the isotopic substitution allows the reliable identification of fragments involving labeling atoms. In both the NBD and ^13^C NBD-modified Au NP samples, the fragment NO_2_^–^ was observed with an intense peak, followed by the fragments C_6_H_4_NO_2_^–^ and ^13^CC_5_H_4_NO_2_^–^, respectively. This confirms the aryldiazonium film as observed by SERS and ^13^C CP/MAS ssNMR spectra (Figure 3A,B). Most importantly, the fragments containing Au–C bonding were identified in the form of Au_2_C_6_H_4_NO_2_^–^ and Au_2_^13^CC_5_H_4_NO_2_^–^ (Figure 3C). The accuracy (|Δ|) of all these assignments is ≤100 ppm, meaning that they are in agreement with the theoretical mass (Table 1). Specifically, the fragments ^13^CC_5_H_4_NO_2_^–^ and Au_2_^13^CC_5_H_4_NO_2_^–^ from the ^13^C NBD sample are exactly 1 u heavier than their corresponding fragments from the NBD sample. These alignments of the mass difference between labeled and unlabeled atoms validate the postulated structures (Table 1). The detection of Au_2_C_6_H_4_NO_2_^–^ and Au_2_^13^CC_5_H_4_NO_2_^–^ provides strong evidence of the grafting of the aryl groups onto the Au surface through Au–C covalent bonding. Thus, we can conclude that the band at 165 ppm in ^13^C NMR can be assigned to Au–C bonds, and the band at 148 ppm can be assigned to the multilayered structure involving aryl moieties bounded to the *ortho*-C of the phenyl ring of the underlying layer (Table S2).

**Table 1:** Tabulation of the Fragments Obtained from NBD-Modified Au NPs and ^13^C NBD-Modified Au NPs in ToF-SIMS^a^

samples	fragment	*M*_meas_ (u)	*M*_ext_ (u)	|Δ| (ppm)
NBD on Au NPs	NO_2_^–^	45.9976	45.9935	90.3
	C_6_H_4_NO_2_^–^	122.0194	122.0248	43.5
	Au_2_C_6_H_4_NO_2_^–^	515.9662	515.9573	16.1
^13^C NBD on Au NPs	NO_2_^–^	45.997	45.9935	76.1
	^13^CC_5_H_4_NO_2_^–^	123.0252	123.0281	23.5
	Au_2_^13^CC_5_H_4_NO_2_^–^	516.9608	516.9612	0.7

a Assignment of the peaks is based on a comparison of the experimentally recorded mass (following mass calibration), *M*_meas_, with that of the theoretical (exact) mass of the candidate fragment ion, *M*_ext_. The accuracy of this assignment is expressed as a parameter, |Δ|, in parts per million, where an assignment is assumed to be correct when |Δ| ≤ 100 ppm (see Methods section).

## Conclusion

In conclusion, unequivocal evidence of the Au–C bond has been validated through ^13^C CP/MAS ssNMR and ToF-SIMS spectroscopy coupled with isotopic substitution of the *ipso*-carbon in nitrobenzenediazonium-modified Au NPs. It is widely accepted that the band observed at 412 cm^–1^ in the SERS spectrum arises from the Au–C covalent bonding. However, upon addressing the relevant ^13^C isotope, this band shows no isotopic shift in the SERS spectrum. This finding indicates that using the 412 cm^–1^ band as evidence of the Au–C bond is, at best, inconclusive. The Au–C bond was subsequently investigated by ^13^C CP/MAS ssNMR, taking advantage of isotopic labeling. Detailed analysis demonstrates that the Au–C junction appears at 165 ppm, while another signal at 148 ppm is attributed to multilayer growth as a result of arylations of already grafted layers via an aryl radical mechanism. Furthermore, the Au–C bonding was confirmed by ToF-SIMS measurements showing fragments of Au_2_C_6_H_4_NO_2_^–^ and Au_2_^13^CC_5_H_4_NO_2_.

The use of ^13^C CP/MAS ssNMR with isotope substitution is particularly advantageous in identifying covalent Au–C bonds resulting from various organic molecules. The confirmation of the covalent sigma Au–C bond fills the gap in metal–C bonds on surfaces, and it opens the possibility for rational exploration and optimization of the highly efficient molecular junction based on the Au–C covalent bond. It will be particularly useful to guide the development of well-defined monolayer modifications from the reduction of diazonium salts on Au surfaces.^42−44^

## Methods

### Au NP Synthesis

#### ^13^C CP/MAS ssNMR

Aqueous solutions of gold(III) chloride hydrate (240 mL, 0.5 mM) and sodium citrate aqueous solution (10 mL, 38.8 mM) were mixed under vigorous stirring for 10 min. Afterward, a freshly prepared sodium borohydride aqueous solution (10 mL, 0.1 M) was added while stirring was continued for 3 h at room temperature (RT), yielding colloidal Au NPs with 8 nm in diameter (Figure S7A). The resulting Au NP colloidal solution was purified by diafiltration (molecular weight cutoff of 5 kDa) for 12 h and concentrated to 120 mL for further use.

#### SERS and FTIR

An aqueous solution of gold(III) chloride hydrate (50 mL, 0.29 mM) was heated to boiling. A sodium citrate aqueous solution (0.21 mL, 38.7 mM) was added. Within 20 min, the gold(III) chloride hydrate was completely reduced,^46^ yielding colloidal Au NPs with 90 nm in diameter (Figure S7B).

Au NPs with 90 nm in diameter have shown the highest enhancement in our SERS experiments. On the other hand, the ^13^C CP/MAS ssNMR study required a high specific surface area to maximize the loading in the organic film (and thus in Au–C bonds) to obtain a sufficient signal-to-noise ratio in the NMR spectra. The best compromise in particle size with respect to loading and ease of separation by centrifugation for purification was 8 nm. To achieve a suitable signal-to-noise ratio, 4 × 10^4^ ssNMR scans were sufficient even for the nonisotopically labeled samples.

### Diazonium Grafting onto Au NPs

#### SERS

A 10 mL volume of Au NPs (90 nm, 7.7 × 10^9^ particles mL^–1^) was mixed with 100 μL of 4-nitrobenzenediazonium tetrafluoroborate (NBD) solution (2.44 mM in DMSO). For the corresponding 4-nitro-[1-^13^C]-benzenediazonium tetrafluoroborate (^13^C NBD) and ^15^N-4-nitrobenzenediazonium tetrafluoroborate (^15^N NBD) samples, the modification procedure was exactly the same. The reagents were left to incubate for 24 h. Afterward, the NPs were separated from the solution via centrifugation at 2000 rpm for 5 min (Rotofix 32A centrifuge). The Au NPs were redispersed in 10 mL of deionized water with ultrasonication for 10 s. This centrifugal process was repeated three times. The sample was finally dried under vacuum at RT.

#### ^13^C CP/MAS ssNMR

Au NPs (120 mL, 8 nm, 3.8 × 10^13^ particles mL^–1^) were mixed with 2 mL of NBD (12.66 mM in acetonitrile). For the corresponding ^13^C NBD and ^15^N NBD samples, the modification procedure was exactly the same. The reagents were left to incubate for 24 h. Afterward, the NPs were separated from the solution via centrifugation at 35 000 rpm for 40 min at 4 °C using an ultracentrifuge. The Au NPs were redispersed in 120 mL of deionized water. This centrifugal process was repeated three times, and the final solid was dried under vacuum at RT.

### Physicochemical Characterization

#### NMR

^1^H NMR spectra were recorded using a Bruker DPX-200 spectrometer. Chemical shifts (δ) are referenced to the solvent signal and given in ppm. Data are reported as follows: chemical shift, multiplicity (s, singlet; d, doublet; t, triplet; m, multiplet), coupling constants (*J*), and integration. ^13^C CP/MAS NMR spectra were collected on a Bruker DSX 400 WB NMR spectrometer operating at 100.57 MHz for ^13^C, with MAS rates of 8 kHz (for sample of NBD on Au NPs) or 12 kHz (for a sample of ^13^C NBD on Au NPs), 90° proton pulse lengths of 5 μs, contact times of 4 ms, and delay times of 3 s (for a sample of ^13^C NBD on Au NPs) or 10 s (for a sample of NBD on Au NPs). A ^1^H spin-locking field of 50 kHz was applied throughout with a 50 kHz ^1^H decoupling field. Chemical shifts (δ) are referenced to adamantane and given in ppm.

#### Raman

Surface-enhanced Raman scattering was recorded with a Jobin–Yvon iHR550 spectrometer (Bensheim, Germany) equipped with a thermoelectric cooled charge coupled device. Excitation was done with a laser MPC 6000, Model Ignis 660 (Laser Quantum, Stockport, UK) at a wavelength of 661 nm. The typical laser power was 10 mW, and the spectra were acquired for 30 s.

#### ToF-SIMS

ToF-SIMS analysis was achieved using a ToF.SIMS 5 (ION-ToF GmbH, Münster, Germany) instrument. A static SIMS condition with a total ion dose of <10^13^ ions cm^–2^ per analysis was employed using a 9.5 keV Bi_3_^+^ primary ion beam. The ion beam operated in the high-current bunched mode for high spectral resolution of >10^4^ at low mass (*m*/*z* = 29 u). Spectra were acquired over a 1–850 u mass range in both positive and negative ion modes. Charge compensation was achieved using a pulsed electron flood source. Fragments of known composition, at low and high masses, were used for mass calibration, including those characteristic of gold and nitrobenzene. The assignment of the peaks is based on a comparison of the experimentally recorded mass (following mass calibration), *M*_meas_, with that of the theoretical (exact) mass of the candidate fragment ion, *M*_ext_. The accuracy of this assignment is expressed as a parameter, |Δ|, in parts per million (eq 1), where an assignment was assumed to be correct when |Δ| ≤ 100 ppm.
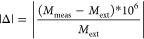
1

#### Dynamic Light Scattering

Dynamic light scattering (DLS) was applied to determine the size and size distribution of gold nanoparticles in colloid solution via a Malvern Zetasizer Nano ZS.

## Supporting Information Available

Experimental details on methods, material synthesis, and characterization (Figure S1–S6, Table S1–S2) (PDF)
