# Supplementary material for: Spectroscopic Evidence for a Covalent Sigma Au–C Bond on Au Surfaces Using 13C Isotope Labeling
Source: JACS Au. 2021 Feb 23;1(3):362–8. doi: 10.1021/jacsau.0c00108 (PMC8016281; doi:10.1021/jacsau.0c00108)
Supplement: Supplementary file 1 — au0c00108_si_001.pdf [file au0c00108_si_001.pdf]

Supplementary Information

# Spectroscopic Evidence for a Covalent Sigma Au−C Bond on Au Surfaces Using $^{13}C$ Isotope Labeling

Huaiguang Li,*[,†,§] Gabriel Kopiec,[†] Frank Müller,[†] Frauke Nyßen,[†] Kyoko Shimizu,[‡] Marcel Ceccato,[‡] Kim Daasbjerg,[‡] and Nicolas Plumeré *[,†,§]

[†]Center for Electrochemical Sciences (CES), Faculty of Chemistry and Biochemistry, Ruhr University Bochum, Universitätsstr. 150, D-44780 Bochum, Germany

[‡]Interdisciplinary Nanoscience Center/Department of Chemistry, Aarhus University, Gustav Wieds Vej 14, 8000 Aarhus C, Denmark

[§]Campus Straubing for Biotechnology and Sustainability, Technical University Munich, Schulgasse 22, 94315 Straubing, Germany

*E-mail: huaiguang.li@rub.de. (H.L.)
*E-mail: nicolas.plumere@tum.de. (N.P.)

**Table of Contents**

**Figure S1:** Raman spectrum of NBD and $^{15}N$ NBD powder.

**Figure S2:** $^{1}H$ NMR of NBD.

**Figure S3:** Raman spectra of NBD powder, NBD modified Au NPs, $^{13}C$ NBD modified Au NPs, and $^{15}N$ NBD modified Au NPs.

**Figure S4:** $^{13}C$ NMR spectra recorded of NBD.

**Table S1:** Peak Assignments for $^{13}C$ NMR of 4-Nitrophenol.

**Figure S5:** Raman spectra of NBD modified Au NPs, 4-nitrophenol, and 4-nitrophenol modified Au NPs.

**Figure S6:** FTIR spectrum of NBD modified Au NPs.

**Table S2:** Peak Assignments for $^{13}C$ NMR of NBD Modified Au NPs.

**Figure S7:** Size distribution of Au NPs.

## Experimental Procedures

**Synthesis of 4-nitrophenol modified Au NPs for Raman measurement**. 10 mL Au NPs (90 nm) was mixed with 100 µL 4-nitrophenol solution (4.16 mM in acetonitrile). The reagents were left to incubate for 24 h. Afterwards, the NPs were separated from solution using centrifugation at 2000 rpm for 5 min (Rotofix 32A centrifuge). The Au NPs were re-dispersed in 10 mL of deionized water with ultrasonication for 10 s. This centrifugal process was repeated 3 times.

**Synthesis of $^{15}$N labeling 4-nitrobenzenediazonium ($^{15}$N NBD)**. 4-Nitroaniline (20 mg, 0.145 mmol) was dissolved in 3 mL tetrafluoroboric acid (48% wt. in $H_2O$) and cooled to 0 °C while stirring. Afterwards, a solution of $^{15}$N labeled sodium nitrite (10 mg, 0.145 mmol) in 24 µL deionized water was added carefully and stirred for further 10 min at RT. The solvent was removed under vacuum and the product dissolved in acetonitrile. After filtration, the solid compound was washed several times with a mixture of petroleum ether/acetone (1:1) to yield the pure product. Yield = 31.6 mg (0.133 mmol, molar mass = 237.92 g mol$^{-1}$) which is equivalent to 92%.

**Synthesis of 4-nitro-[1-$^{13}$C]-benzenediazonium ($^{13}$C NBD)**[S1]

***a) 4-nitro[1-$^{13}$C] phenol*****.** Sodium-nitromalonaldehyde (254 mg, 1.62 mmol) was dissolved in 31.3 mL of NaOH (131.46 mmol, 4.2 M) and cooled with an ice/sodium chloride bath. To this, 0.1 mL of [2-$^{13}$C] acetone (81.51mg, 1.38 mmol) in 3.91 mL of NaOH (16.42 mmol, 4.2 M) was added dropwise over 30 min while stirring. The solution was stirred for additional 140 min at ice bath temperature. After that, the temperature was slowly raised to RT. After stirring for additional 2 h at RT the mixture was acidified with 13.56 mL HCl (162 mmol, 12 M) which was added dropwise over 1 h while cooling the reaction mixture using an ice bath. 100 mL diethylether was added to the mixture which was stirred vigorously for 24 h at RT. The organic layer was dried over $MgSO_4$ and the solvent was removed under reduced pressure. The crude product was first purified by sublimation (180–200 °C) of the impurities using a bulb-to-bulb distillation under vacuum and then stirred in deionized water/diethylether in the presence of charcoal. After separation of the phases the organic layer was dried with $MgSO_4$ and the solvent removed under reduced pressure to give yellow crystals. Yield = 60 mg (0.428 mmol, molar mass = 140.11 g mol$^{-1}$) which is equivalent to 31%. $^{1}$H NMR (200 MHz, $CDCl_3$): $\delta$ (in ppm) = 8.18 (dt, $J$ = 2.5, 9.3 Hz, 2H), 6.93 (dd, $J$ = 2.5, 9.3 Hz, 2H), 6 (s, 1H). $^{13}$C NMR (50 MHz, $CDCl_3$): $\delta$ (in ppm) = 161.53, 126.46, 116.50, 115.19.

***b) 4-(4-nitro-[1-$^{13}$C] phenoxy)-2-phenylquinazoline*****.**14 mL dry acetone was purged with argon for 30 min. After adding dry $K_2CO_3$ (148 mg, 1.07 mmol), 4-chloro-2-phenylquinazoline (107.8 mg; 0.448 mmol) and 4-nitro[1-$^{13}$C] phenol (60 mg, 0.428 mmol) the solution was purged with argon for additional 10 min. The mixture was refluxed under argon for 48 h. After cooling to RT, 20 mL iced deionized water was added to the mixture and the product precipitated from the solution. Stirring was performed for additional 30 min. The solid was filtrated, washed with cold deionized water (35 mL) and dried in vacuum. Yield =

145 mg (0.421 mmol, molar mass = 344.4 g $mol^{-1}$) which is equivalent to 98.4%.$^{1}H$ NMR (200 MHz, $CDCl_3$): $\delta$ (in ppm) = 8.47 (s, 1H), 8.42 (d, $J$ = 1.0 Hz, 2H), 8.39–8.26 (m, 8H), 8.10 (d, $J$ = 8.4 Hz, 2H), 7.94 (ddd, $J$ = 8.4, 7.0, 1.4 Hz, 2H), 7.69 (d, $J$ = 1.1 Hz, 1H), 7.65 (t, $J$ = 1.1 Hz, 1H), 7.58 (dd, $J$ = 9.2, 3.8 Hz, 5H), 7.45 (dd, $J$ = 5.1, 1.9 Hz, 6H). $^{13}C$ NMR (50 MHz, $CDCl_3$): $\delta$ (in ppm) = 157.70, 134.68, 128.72, 128.50, 127.50, 125.48, 123.64, 123.43, 122.27, 77.16.

***c) Synthesis of 4-nitro-[1-$^{13}$C] aniline*.** 4-(4-nitro-[1-$^{13}$C] phenoxy)-2-phenylquinazoline (145 mg, 0.421 mmol) was heated under argon atmosphere (300 °C) for 90 min. The increase of the carbonyl signal was followed by IR spectroscopy to make assure that the conversion would be complete. The brown residue (134 mg) was dissolved in a mixture of 10 mL MeOH with1 mL deionized water and purged for 10 min with argon. After adding solid KOH (390 mg, 6.95 mmol) the mixture was stirred under reflux overnight. After cooling down to RT and acidifying with 0.5 mL HCl (6 mmol, 12 M), the mixture was stirred for additional 1.5 h at 50 °C. Afterwards, the mixture was cooled using an ice bath and made alkaline with 50% (w/v) aqueous KOH. The solvent was removed under reduced pressure. The precipitate was dissolved in 30 mL deionized water and extracted with diethylether (3 × 20 mL, 1 × 25 mL). The organic layer was dried over $MgSO_4$ and the solvent was removed under reduced pressure. The crude product was purified by sublimation using the bulb-to-bulb distillation under vacuum (220 °C). Yield = 44 mg (0.316 mmol; molar mass 139.12 g $mol^{-1}$) which is equivalent to 75%.

***d) 4-nitro-[1-$^{13}$C]-benzenediazonium tetrafluoroborate.*** 4-nitro-[1-$^{13}$C] aniline (15 mg, 0.108 mmol) was dissolved in a mixture of 2 mL deionized water with 0.218 mL $HBF_4$ and cooled to 0 °C in an ice bath. 0.3 mL of $NaNO_2$ solution (0.03 mmol, 0.1 M) was added and stirred for 10 min. After warming up to RT, the solution was stirred for another 10 min and the solvent was lyophilized. The crude product was taken up in acetonitrile and filtered through a pipette filter. After the addition of cold diethyl ether, the white crystals were collected as pure product. Yield = 22.9 mg (0.096 mmol, molar mass 237.92 g $mol^{-1}$) which is equivalent to 89%.

**FTIR.** FTIR spectra were performed using a TENSOR 27 FTIR from Bruker (Billerica, USA). Each measurement comprised 250 scans. The spectra were acquired with a liquid nitrogen cooled mercury cadmium telluride detector in the wavenumber range of 4000–600 $cm^{-1}$. The sample was prepared as follows: 10 mL of 90 nm Au NPs was mixed with 100 µL of 4-nitrobenzenediazonium tetrafluoroborate (NBD) solution (2.44 mM in DMSO). The reagents were left to incubate for 24 h. Afterwards, the NPs were separated from solution via centrifugation at 2000 rpm for 5 min (Rotofix 32A centrifuge). The Au NPs was re-dispersed in 10 mL deionized water with ultrasonication for 10 s. This centrifugal process was repeated 3 times. The sample was finally dried under vacuum at RT.

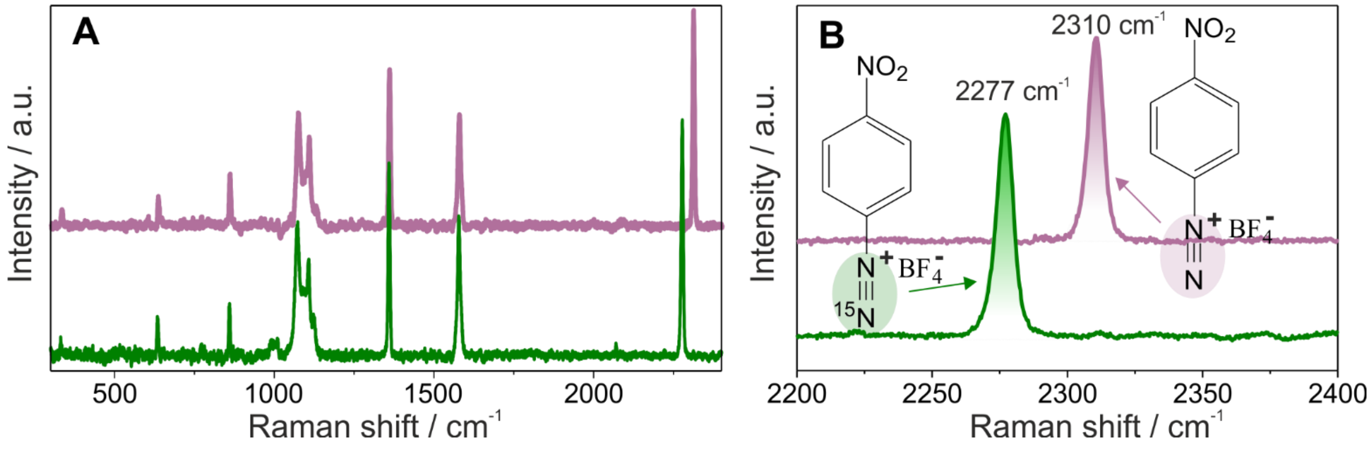


**Figure S1.** A) Raman spectrum of NBD (purple) and $^{15}N$ NBD (green) powder. B) Comparison of N≡N stretching vibrations in $^{15}N$ NBD and NBD.

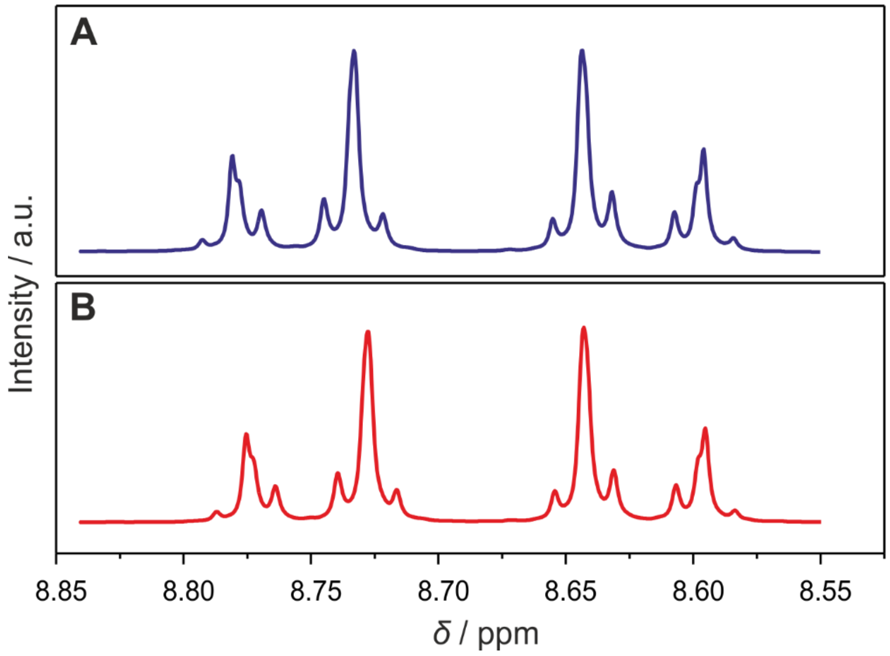


**Figure S2.** $^{1}H$ NMR of freshly dissolved A) NBD in $CD_3CN$ and B**)** after incubating 24 h in acetonitrile.

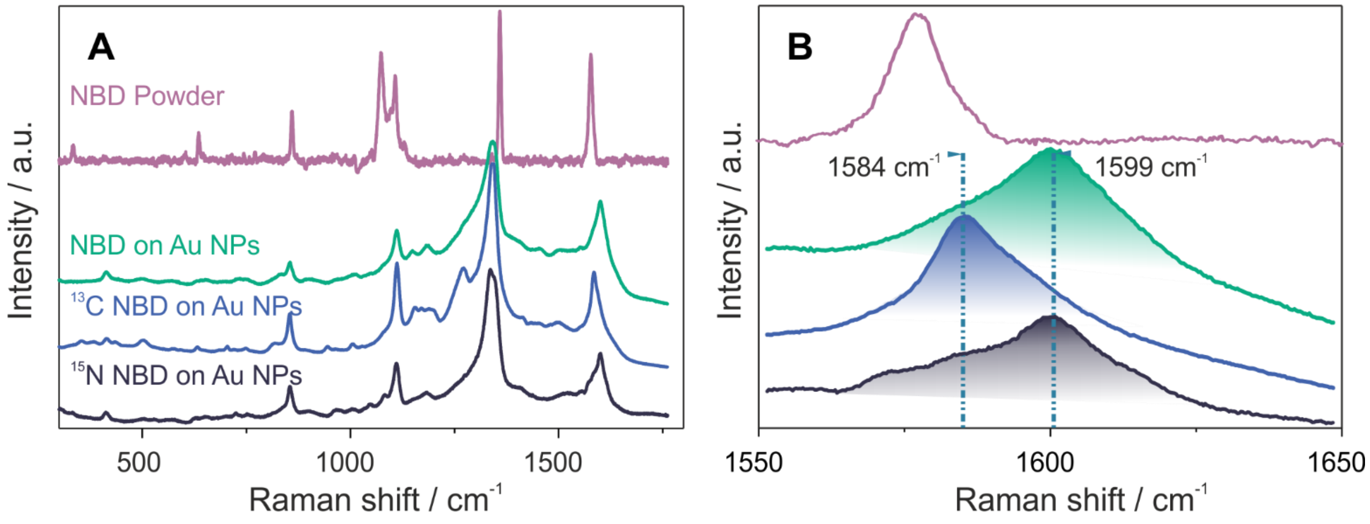


**Figure S3.** Raman spectra of NBD powder, NBD modified Au NPs, $^{13}C$ NBD modified Au NPs, and $^{15}N$ NBD modified Au NPs with A) full Raman spectra and B) spectra range from 1550–1650 $cm^{-1}$.

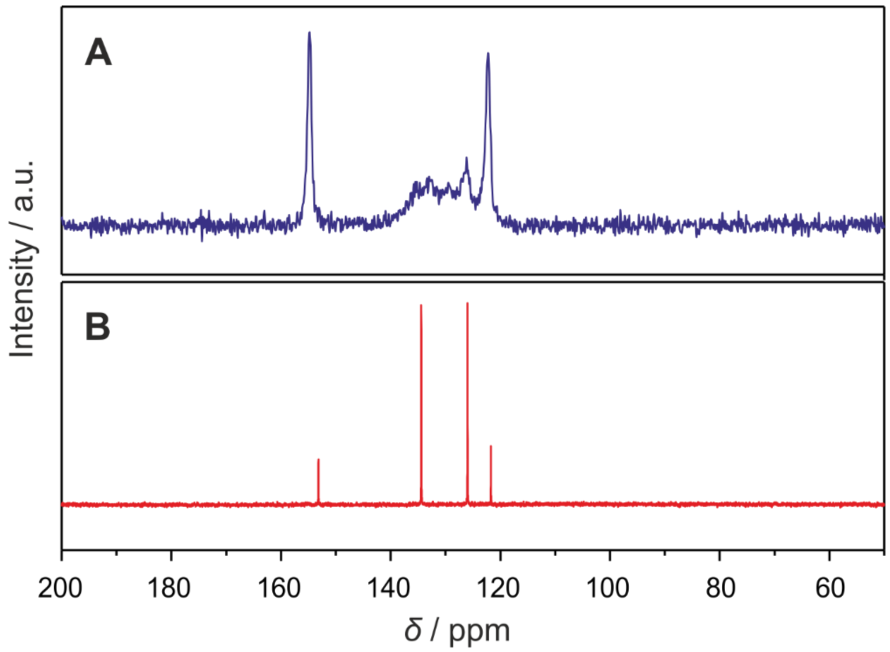


C

| Carbon atoms | Assignment | *δ* (ppm) |
|---|---|---|
| C 1 | ipso C | 121 |
| C 2 | ortho C | 137 |
| C 3 | meta C | 125 |
| C 4 | para C | 155 |

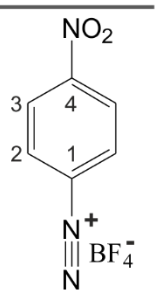


**Figure S4.** $^{13}C$ NMR spectra recorded of NBD in A) solid state and in B) liquid state dissolved in DMSO-$d_6$. C) Peak assignments listed along with the chemical structure demonstrating the position of carbon atoms.

**Table S1.** Peak Assignments for $^{13}$C NMR of 4-Nitrophenol.[a]

| Carbon atoms | δ (ppm) |
|---|---|
| C 1 | 164 |
| C 2 | 116 |
| C 3 | 126 |
| C 4 | 141 |

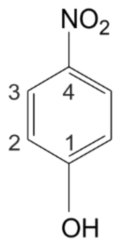


[a] Position of carbon atoms is indicated in the chemical structure.

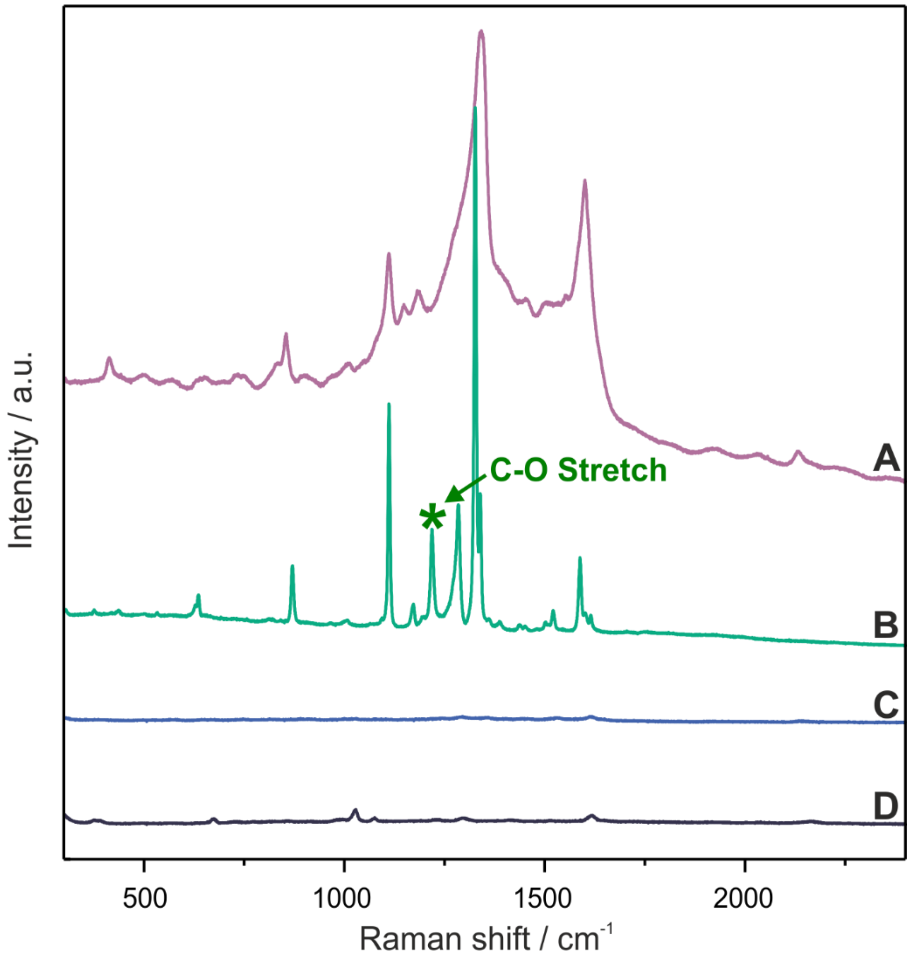


**Figure S5.** Raman spectra of A) NBD modified Au NPs, B) 4-nitrophenol, C) 4-nitrophenol modified Au NPs followed by washing/centrifugation with deionized water, and D) bare Au NPs.

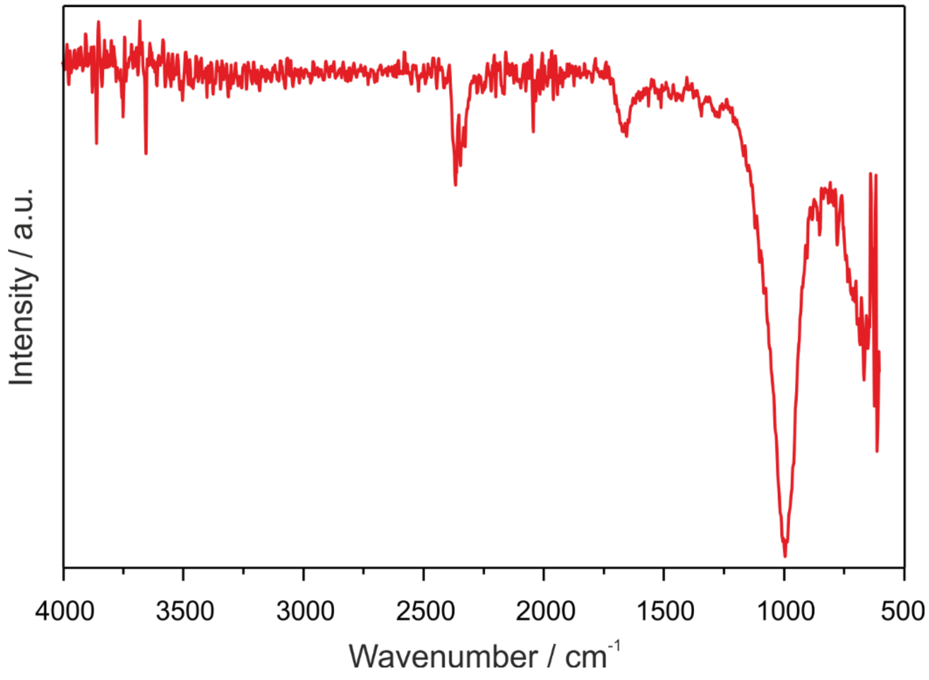


**Figure S6.** FTIR spectrum of NBD modified Au NPs.

**Table S2.** Peak Assignments for $^{13}C$ NMR of NBD Modified Au NPs.[a]

| Carbon atoms | Assignment | δ (ppm) |
|---|---|---|
| C 1 | C-Au | 165 |
| C 2 | ortho C | 137 |
| C 3 | meta C | 125 |
| C 4 | para C | 155 |
| C 5 | multilayer C | 148 |

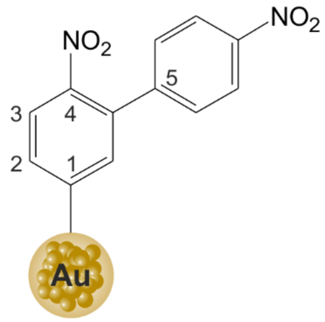


[a] Position of carbon atoms is indicated in the formula structure.

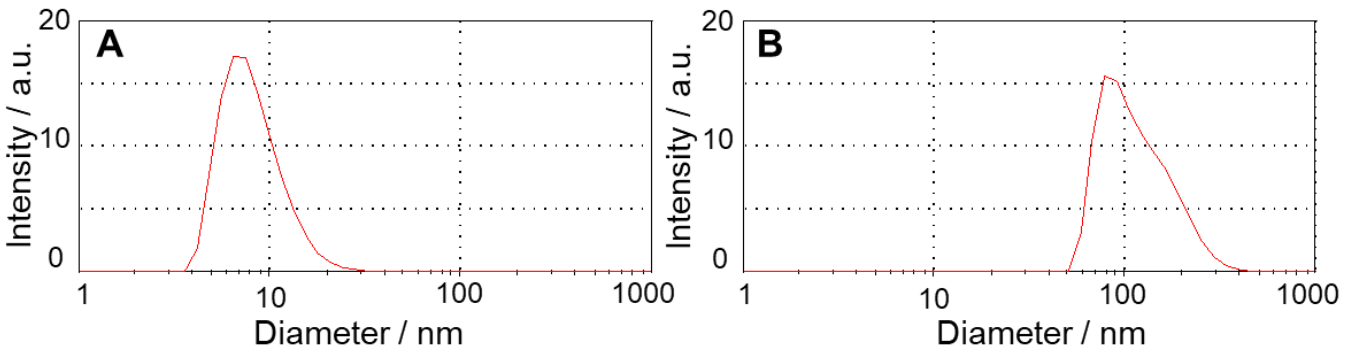


**Figure S7.** Size distribution of Au NPs A) 8 nm in diameter for $^{13}C$ CP/MAS ssNMR and B) 90 nm in diameter for SERS. The size and distribution were measured by dynamic light scattering in a colloid solution using a Malvern Zetasizer Nano ZS.

## References

(S1) Yilmaz, I.; Shine, H. J. Improved yields in the synthesis of 4-nitro[1-$^{13}C$]aniline. *J. Label. Compd. Radiopharm.* **1989**, *27*, 763–766.
